# Electrochemically Reduced Water Protects Neural Cells from Oxidative Damage

**DOI:** 10.1155/2014/869121

**Published:** 2014-10-14

**Authors:** Taichi Kashiwagi, Hanxu Yan, Takeki Hamasaki, Tomoya Kinjo, Noboru Nakamichi, Kiichiro Teruya, Shigeru Kabayama, Sanetaka Shirahata

**Affiliations:** ^1^Department of Bioscience and Biotechnology, Faculty of Agriculture, Kyushu University, Fukuoka 812-8581, Japan; ^2^Division of Life Engineering, Graduate School of Systems Life Sciences, Kyushu University, 6-10-1 Hakozaki, Higashi-ku, Fukuoka 812-0053, Japan; ^3^Nihon Trim Co. LTD., 1-8-34 Oyodonaka, Kita-ku, Osaka 531-0076, Japan

## Abstract

Aging-related neurodegenerative disorders are closely associated with mitochondrial dysfunction and oxidative stresses and their incidence tends to increase with aging. Brain is the most vulnerable to reactive species generated by a higher rate of oxygen consumption and glucose utilization compared to other organs. Electrochemically reduced water (ERW) was demonstrated to scavenge reactive oxygen species (ROS) in several cell types. In the present study, the protective effect of ERW against hydrogen peroxide (H_2_O_2_) and nitric oxide (NO) was investigated in several rodent neuronal cell lines and primary cells. ERW was found to significantly suppress H_2_O_2_ (50–200 *μ*M) induced PC12 and SFME cell deaths. ERW scavenged intracellular ROS and exhibited a protective effect against neuronal network damage caused by 200 *μ*M H_2_O_2_ in N1E-115 cells. ERW significantly suppressed NO-induced cytotoxicity in PC12 cells despite the fact that it did not have the ability to scavenge intracellular NO. ERW significantly suppressed both glutamate induced Ca^2+^ influx and the resulting cytotoxicity in primary cells. These results collectively demonstrated for the first time that ERW protects several types of neuronal cells by scavenging ROS because of the presence of hydrogen and platinum nanoparticles dissolved in ERW.

## 1. Introduction

Living organisms inevitably live with the process of aging which has been closely linked as a critical causative factor for the development of neurodegenerative disorders (NDs) such as Alzheimer's disease (AD), Parkinson's disease (PD), Huntington's disease (HD), and Amyotrophic lateral sclerosis (ALS) [1–3]. Moreover, the development of aging-related NDs has been shown to be closely associated with mitochondrial dysfunction and oxidative stresses [1, 4, 5]. Oxidative stress is a state where the cell is overwhelmed with reactive oxygen species (ROS) and reactive nitrogen species (RNS), resulting from an imbalance between reactive species production and detoxification [3, 6]. Aerobic organisms absolutely require oxygen for the maintenance of normal physiological functions. In particular, the brain is responsible for more than 20% of total body oxygen consumption and 25% of total body glucose utilization although it represents less than 2% of the whole body weight [7]. Such high oxygen consumption in the brain consequently produces a large amount of reactive species compared to other organs in the body [6]. ROS include a variety of diverse chemical species such as superoxide anion (O_2_^•−^), hydroxyl radical (^•^OH), and hydrogen peroxide (H_2_O_2_), all of which are capable of causing extensive oxidative damage to biological macromolecules like DNA, RNA, proteins, and lipids [8–10]. In addition, RNS such as nitric oxide (NO, an unstable free radical) is induced by glutamate, a neurotransmitter abundant in the central nervous system (CNS) and essential for proper neurological processes. However, NO in excess leads to pathological complications such as NDs. Accumulated data have shown that glutamate induces Ca^2+^ influx by way of the glutamate receptor channel, N-methyl-D-aspartate (NMDA). The increased intracellular Ca^2+^ levels together with endogenous calmodulin stimulate nitric oxide synthase (NOS) to produce NO which is converted to neurodestructive NO radical (NO^•^) depending on the redox conditions in the neuronal cells. NO^•^ further reacts with O_2_^•−^ generating a potent oxidant, ONOO^−^. Thereafter, ONOO^−^ is rapidly converted to highly reactive ^•^OH which is cytotoxic [2, 10–12]. Moreover, the damage is exasperated by the fact that the CNS contains a high amount of polyunsaturated fatty acids which are readily peroxidized by toxic reactive species [10] and has relatively low levels of antioxidative enzymes [8, 10]. Therefore, such inherent characteristics of the CNS and the ROS and RNS individually or in combination contribute to the development of NDs. Antioxidants, metal chelators, or other agents that improve endogenous enzymatic and nonenzymatic cellular defense systems have been suggested as treatments for NDs [6]. Control of ROS production by mitochondrially targeted antioxidant treatment is expected to delay aging [1]. Antioxidant therapies using plant extracts from seeds and leaves are reported to be beneficial [13] while some other prospective substances have been tested with unsatisfactory results [2, 14]. For antioxidant therapies to be effective, they must cross the blood brain barrier (BBB), which protects the brain from toxins and reduces the bioavailability of exogenous antioxidants in the brain [2, 6]. Therefore, it is highly desirable to develop alternative agents that are able to pass through the BBB and exert antioxidative effects in the brain.

One promising candidate is electrochemically reduced water (ERW) because it has the potential to cross the BBB. ERW produced near the cathode during electrolysis of 2 mM NaOH model water has characteristics of high pH, low dissolved oxygen, and an extremely negative redox potential [15]. ERW contains a high concentration of dissolved hydrogen (0.4–0.9 ppm) and a small amount of platinum nanoparticles (Pt nps, 0.1–2.5 ppb), both of which exhibit ROS-scavenging ability [11, 16, 17]. Neutralized ERW has been shown to exhibit H_2_O_2_-scavenging activity which is correlated with protection of DNA [18, 19] and carbon tetrachloride induced liver damage [20], lifespan extension of* Caenorhabditis elegans* [16, 21], alloxan-induced type 1 diabetes [15, 19], hemodialysis-induced oxidative stress during end-stage renal disease [22, 23], and inhibitory effects of HT1080 tumor cell invasion [24]. ERW, in combination with glutathione, induced human leukemia HL-60 apoptotic cell death whereas a cytotoxic effect was not observed in normal peripheral blood mononuclear cells [25]. Despite the various protective functions exhibited by ERW, its effect on neuronal cells has not been disclosed in the literature other than in a brief meeting abstract by Yan et al. [26] who reported the protective effect of ERW on H_2_O_2_-induced cultured N1E-115 neuroblastoma cell death.

Cultures of nervous system tissues and cells are categorized in the terms of their complexities: whole-embryo, whole brain, organotypic slices, reaggregate cultures, dissociated primary cell cultures, and cell lines [27]. The degree of complexity of an* in vitro* model of dissociated primary cell cultures is considered to more closely reflect the* in vivo* state than that of the cell lines [27]. In light of this view, we also used mouse cerebral cortex neuronal primary (MCCNP) cells as a model to observe the effect of ERW in addition to immortalized cell lines. N1E-115 cells have been established as an adrenergic clone derived from mouse neuroblastoma C-300 [28] and are used as a model of CNS neurons [29–32]. In addition, in culture in the presence of several factors including DMSO, these cells display morphological characteristics of neuritogenesis which we used as a marker for changes upon treatment with ERW [33]. The PC12 cell line was established from a transplantable rat adrenal pheochromocytoma based on its response to nerve growth factor (NGF). PC12 cells possess the potential to be differentiated into either chromaffin cells or sympathetic neurons when in the presence of NGF [34]. This cell line has been used as a model for studying the neuronal response to oxidative stress [35–37]. Also, the viability of PC12 cells is described to be sensitive to NO stress, thus this makes them useful for detecting a subtle NO effect [38]. Serum-free mouse embryo (SFME) cells were established from mouse embryo cells by maintenance in the absence of serum [39]. These cells show the characteristics of an astrocyte, a progenitor cell without senescence which is the most abundant cell type in the CNS [39, 40].

In the present study, we utilized various cell types originating from mouse and rat as a first step to explore the protective effect of ERW on neurocytotoxicity caused by reactive species.

## 2. Materials and Methods 

### 2.1. Materials

Dulbecco's Modified Eagle's Medium (DMEM) and DMEM/Ham's F12 Combined Medium (1 : 1) were purchased from Nissui Pharmaceutical Co., LTD. (Tokyo, Japan). Insulin, putrescine, transferrin, propidium iodide (PI), Fluo-3/AM, pluronic F127, sodium glutamate, and Ca2+, Mg2+-free Hank's balanced salt solution (Ca2+, Mg2+-free HBSS) were purchased from Sigma-Aldrich Japan (Tokyo, Japan). 2′, 7′-Dichlorofluorescin diacetate (DCFH-DA) was purchased from Invitrogen Technologies (Carlsbad, CA, USA). Chemically defined lipid (CDL) and mouse epidermal growth factor (mEGF) were purchased from Life Technologies Japan (Tokyo, Japan). Cell counting kit-8 (CCK-8) which uses WST-8 as a color indicator to measure live cell numbers was purchased from Dojindo Laboratories Co. (Tokyo, Japan) and the kit is referred to as the WST-8 kit hereafter. Diaminorhodamine-4M acetoxymethyl ester (DAR-4M AM) was from Daiichi Pure Chemicals Co., LTD. (Tokyo, Japan). N-Acetyl-L-cysteine (L-NAC), ascorbic acid (AsA), sodium nitroprusside (SNP), 4-[2-hydroxyethyl]-1-piperazineethane-sulfonic acid (HEPES), fetal bovine serum (FBS), bovine serum albumin (BSA), penicillin, streptomycin, progesterone, and all other chemicals were obtained from Wako Pure Chemical Industries, LTD. (Osaka, Japan). The gelatin sepharose 4B column was obtained from GE Healthcare Japan (Tokyo, Japan). Ultrapure water (MQ-water) was produced by a Millipore filtration system (Billerica, MA, USA).

### 2.2. Preparation of ERW

ERW was prepared by electrolysis of MQ-water containing 2 mM NaOH at 100 V for 60 min using a TI-200 electrolysis device (Nihon Trim Co., Osaka, Japan). The device is a batch-type system composed of a 4-liter electrolysis vessel which is divided into two compartments by a semipermeable membrane. Each compartment contains a platinum-coated titanium (Ti) electrode. Further details of the device and the characteristics of ERW including pH, dissolved hydrogen/oxygen and Pt nanoparticle concentrations, and redox potential are given in our previous reports [15, 16, 41]. ERW was neutralized with HEPES buffer or bicarbonate buffer in medium before use. NaOH solution was adjusted to the same pH with that of freshly prepared ERW and used as control water.

### 2.3. Preparation of Cell Culture Medium

5x DMEM/Ham's F12 medium (1 : 1 mixture of 5x DMEM and 5x Ham's F12 medium, no FBS) was diluted by quadruple neutralized ERW or MQ-water as the control to make the control medium. Normal DMEM/F12 medium supplemented with different concentrations of L-NAC or AsA was used as positive controls in this study.

### 2.4. Cell Cultures

Murine neuroblastoma N1E-115 cells were purchased from ATCC (VA, USA) and maintained in 90% DMEM/Ham's F12 medium supplemented with 10% FBS, 2 mM glutamine, 100 U/mL penicillin, and 100 *μ*g/mL streptomycin and incubated in a humidified atmosphere of 5% CO_2_ at 37°C. For differentiation, a previously published method was followed with modifications [31]. N1E-115 cells were cultured in 88% DMEM/Ham's F12 medium containing 10% FBS, 2% dimethyl sulfoxide (DMSO) in poly-D-lysine (PDL)-coated dishes for 6 days, and then the medium was replaced with serum-free DMEM/F12 medium supplemented with 5 *μ*g/mL insulin, 100 *μ*M putrescine, 20 nM progesterone, and 5 *μ*g/mL transferrin.Rat pheochromocytoma PC12 cells were purchased from RIKEN Cell Bank (Tsukuba, Japan). Cells were cultured in DMEM containing 5% horse serum (HS), 5% fetal bovine serum (FBS), 100 U/mL penicillin, and 100 *µ*g/mL streptomycin and incubated in a humidified atmosphere of 5% CO_2_ at 37°C. Medium was changed every 2 days. Differentiation into neuronal cells was carried out by culturing in PDL-coated dishes containing DMEM supplemented with 0.5% HS, 0.5% FBS, and 20 nM nerve growth factor (NGF) for 4 days in a humidified atmosphere of 5% CO_2_ at 37°C. Serum-free mouse embryo (SFME) cells were kindly provided by Loo et al. [39] and cultured in a 1 : 1 mixture of DMEM : Ham's F12 medium containing 10 nM sodium selenite, 1.2 g/L sodium bicarbonate, 10 *μ*g/mL transferrin, 1% of CDL, and 50 ng/mL mEGF in fibronectin-coated 25 cm^2^ T-flasks. Fibronectin was purified from BSA using a gelatin sepharose 4B column by the published method [42].

### 2.5. Preparation of Mouse Cerebral Cortex Neuronal Primary (MCCNP) Cells

MCCNP cells were prepared from the cerebral cortex of fetal mouse (15-16 days gestation) that has been removed aseptically from an anesthetized and decapitated mouse (ddy strain, Asekku-Yoshitomi Co. Fukuoka, Japan). The embryos were removed under sterile conditions and placed into an ice-cold 1 : 1 mixture of phosphate-buffered saline (PBS) and DMEM. Cerebral cortex excised from the brain was minced and incubated for 15 min in a mixture of 0.05% trypsin and PBS. Single cells dissociated from the cerebral cortex were counted, adjusted to 0.5–1.0 × 10^5^ cells/mL, with DMEM supplemented with 5% HS, 5% FBS, 100 U/mL penicillin, and 100 *µ*g/mL streptomycin, and seeded on PDL-coated 24-well plastic plates. Cells were incubated for 2 days in a humidified atmosphere of 5% CO_2_ at 37°C. After the incubation, 10 *µ*M cytosine *β*-D-arabinofuranoside hydrochloride (Ara-C), a deoxyribonucleic acid synthesis inhibitor, was added and the cells were incubated for further 24 h to eliminate rapidly growing nonneuronal cells [43, 44]. Ara-C containing medium was replaced with DMEM growth medium as described above, and the cells were incubated for 7 to 10 days prior to subsequent experiments.

### 2.6. Animal Handling

Mice were handled with care following the guidelines for Animal Experiments provided by the Faculty of Agriculture and the Graduate School of Kyushu University and the Law (No. 105) and Notification (No. 6) of the Government.

### 2.7. Cell Viability Assay

A WST-8 kit was used to obtain a viable cell count (Dojindo Co., Tokyo, Japan). The assay protocol provided by the manufacturer was closely followed. Briefly, N1E115 cells (10^4^ cells/well) were seeded in a PDL-coated 24-well plate and pr-incubated with FBS/DMEM medium. Following cell differentiation with DMSO, cells were treated with serum-free medium made of MQ-water, ERW, L-NAC, AsA, or 2 mM NaOH supplemented with or without different concentrations of H_2_O_2_ for 24 h. To each well, 1 *μ*L of WST-8 dye was added and incubation was continued for further 4 h. Viable cells were measured at 450 nm using a microtiter plate reader (Lancraft, Inc., Norcross, GA, USA). PC12 cells (10^4^ cells/well) were seeded in PDL-coated 24-well plates and preincubated in FBS/HS/DMEM medium. Differentiation was accomplished by adding NGF followed by a 4 day incubation (detail is given in the cell culture section). When cells were differentiated, they were treated with serum-free medium containing ERW with or without different concentrations of H_2_O_2_ or SNP for 24 h. Viable cells were measured as described above. SFME cells (10^5^ cells/well) were seeded in 24-well plates and incubated in the presence of FBS/HS/DMEM medium for 1 day. Cells were treated with serum-free medium containing ERW with or without different concentrations of H_2_O_2_ for 24 h (detail is given in the cell culture section). Viable cells were measured as described above.

### 2.8. Intracellular H_2_O_2_ Detection Using a DCFH-DA Probe

2′, 7′-Dichlorofluorescin diacetate (DCFH-DA) can diffuse into cells where it is hydrolyzed by intracellular esterases resulting in the formation of DCFH. Then, DCFH is oxidized by intracellular oxidizing species including H_2_O_2_ to form the highly fluorescent product, 2′, 7′-dichlorofluorescin (DCF), which can be detected by appropriate devices such as a flow cytometer and/or confocal laser microscope. DCFH-DA has been shown to be useful to estimate intracellular H_2_O_2_ in rat neuron cells [45].

### 2.9. Flow Cytometric Analysis of Intracellular H_2_O_2_

N1E-115 cells (7.5 × 10^4^ cells) were seeded in an uncoated 10 cm dish and precultured for 1 day. Then, the medium was replaced with HBSS (137 mM NaCl, 5 mM KCl, 1 mM Na_2_HPO_4_, 5 mM glucose, 1 mM CaCl_2_, 0.5 mM MgCl_2_, 1 mg/mL BSA, and 10 mM HEPES, pH 7.4) buffer containing ERW, L-NAC (0.1, 1.0 mM), AsA (0.1, 1.0 mM), or control MQ-water and was incubated for 10 min. After removing the supernatant, 2 mL of 5 *μ*M DCFH-DA, in Ca^2+^, Mg^2+^-free HBSS, adjusted pH to 7.4 with 10 mM HEPES buffer, was added to the dish and incubated for further 15 min. Cells were then harvested in 5 mL of PBS buffer and collected by centrifugation at 500 ×g for 3 min and then resuspended in 1 mL of PBS. Fluorescence intensities were measured immediately using an EPICS XL System II-JK flow cytometer (Beckman Coulter, Miami, USA), with excitation and emission wavelengths of 495 and 525 nm, respectively. Histograms were analyzed by using FlowJo software supplied with the cytometer.

### 2.10. Confocal Laser Microscopic Analysis of Intracellular H_2_O_2_

The intracellular H_2_O_2_ was visualized using the DCFH-DA probe. One milliliter of N1E115 cells (2 × 10^4^ cells/mL) was precultured in a 35 mm dish for 1 day. Then, the medium was replaced with HBSS made with ERW or control MQ-water, with or without 200 *μ*M of H_2_O_2_, and incubated for 10 min. After removing the supernatant, 2 mL of 5 *μ*M DCFH-DA in Ca^2+^, Mg^2+^-free HBSS, adjusted to pH 7.4 with 10 mM HEPES buffer, was added and the dish was incubated for further 10 min. Cells were then washed with HBSS to remove unreacted DCFH-DA and the cytoplasmic fluorescence intensities were measured using a confocal laser scanning microscope (Molecular Dynamics, Sunnyvale, CA, USA) with a FITC barrier filter at excitation and emission wavelengths of 488 and 530 nm, respectively.

### 2.11. Assessment of Nitric Oxide (NO) Toxicity Using a Cell Viability Assay

Differentiated PC12 cells were cultured in a medium made of ERW or MQ-water each containing 0, 200, 400, or 600 *μ*M sodium nitroprusside (SNP) for 24 h. The viability of the cells was measured using the WST-8 kit method.

### 2.12. Detection of Intracellular NO

Intracellular NO in the cells can be detected using a rhodamine chromophore, the DAR-4M AM NO bioimaging probe. DAR-4M AM diffuses into cells and is hydrolyzed by intracellular esterases resulting in the formation of membrane impermeable DAR-4M. DAR-4M then reacts with NO in the presence of oxygen forming a fluorescent triazol compound, DAR-4M T, which can be visualized using a fluorescence microscope at 560 nm excitation and 575 nm emission [46]. For PC12 cells, 2 × 10^5^ cells were seeded in a 35 mm dish and incubated for 1 day. Cells were then treated with the medium made of ERW or MQ-water each containing 200 *μ*M SNP for 20 min. Following the incubation period, cells were washed once with HBSS and 10 *μ*M DAR-4M AM in HBSS was added and the cells were incubated for 30 min. The cells were washed once with HBSS and intracellular fluorescence was observed using a fluorescence microscope (IX70, Olympus, Japan) and was photographed.

### 2.13. Measurement of Intracellular Ca^2+^

Intracellular Ca^2+^ was measured using Fluo-3 AM, a membrane-permeable calcium sensitive dye. Internalized Fluo-3 AM is hydrolyzed by intracellular esterases liberating Flou-3 which can react with intracellular free Ca^2+^ ions to form a fluorescent complex. After pretreatment with glutamate (0, 0.1 or 1.0 mM) in medium made of ERW or MQ-water for 10 min, PMN cells were stained with 300 *μ*L of dye mixture composed of 4 *µ*M Fluo-3 AM and 0.8 mg/mL pluronic F127 in HBSS, pH 7.4 for 20 min, and then 1 mL of 1% FBS containing HBSS was added followed by a further 40 min incubation [47]. Cells were washed once with HBSS and suspended in 1 mL HEPES buffer. A fluorescence microscope was used to detect intracellular fluorescence and the images were recorded by a digital camera. Digital images were converted to a numerical readout with the aid of a NIH image analysis program and then were analyzed by Excel software.

### 2.14. Measurement of Glutamate Neurotoxicity

Glutamate toxicity was measured using PMN cells. After cells were treated with 0, 0.1, and 0.5 mM glutamate in the medium made of ERW or MQ-water for 24 h, nonviable cells were detected using trypan blue dye which does not enter live cells because of their intact cell membranes. Treated cells were stained with 0.1% trypan blue for 10 min, fixed with 4% paraformaldehyde for 15 min at 4°C, and rinsed once with PBS. The nonviable cells stained blue by the dye were counted in 8 randomly selected fields as one set and 4 sets for each treatment were counted under the microscope (CK2, Olympus, Japan).

### 2.15. Statistical Analysis

Three to four independent experiments were performed. Data are presented as the mean ± standard deviation (SD). Statistical significance was determined by repeated measures analysis of variance with* post hoc* comparisons using the Tukey test and a *P* value of < 0.05 was considered significant.

## 3. Results

### 3.1. ERW Protects Neural Cells From H_2_O_2_-Induced Cell Death

**Figure 1:**
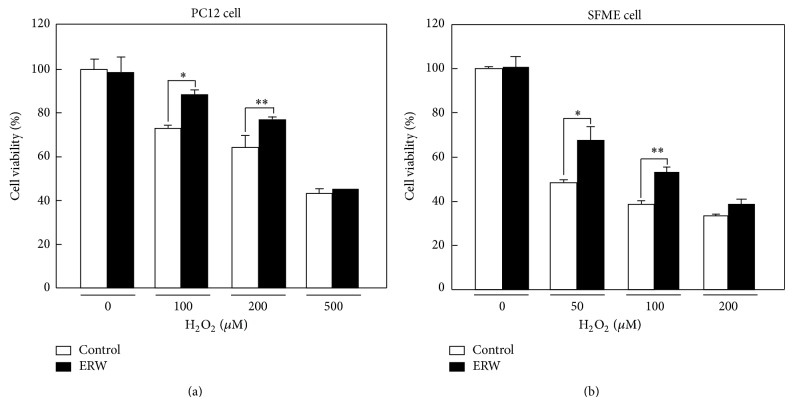
Suppressive effect of ERW against H_2_O_2_-induced cytotoxicity on PC12 and SFME cells. Viable cell numbers were measured using a WST-8 kit. (a) Pheochromocytoma PC12 cells were cultured in FBS/HS/DMEM medium with or without ERW and the number of surviving cells were set as controls. H_2_O_2_ (100, 200 and 500 *μ*M) was added to the control medium and it was cultured for 24 h. Viable cells were measured at 450 nm using a microtiter plate reader. (b) Serum-free mouse embryo (SFME) cells were cultured in a 1 : 1 mixture of DMEM : Ham's F12 medium with or without ERW and the number of surviving cells were set as controls. To the control medium, H_2_O_2_ (50, 100 and 200 *μ*M) was added and it was cultured for 24 h. Viable cells were measured at 450 nm using a microtiter plate reader. Data are presented as mean ± standard deviation (SD) for three independent experiments (*n* = 3 each set). ^*^ *P* < 0.05 and ^**^ *P* < 0.01.

ERW has been reported to suppress H_2_O_2_-induced oxidative stress in neuroblastoma N1E115 cells [26]. We first examined whether H_2_O_2_-induced oxidative stress was suppressed by ERW in two other neuronal cell lines, PC-12 and SFME. Because PC12 and SFME cells showed different sensitivity to H_2_O_2_ in terms of cell viability, different H_2_O_2_ concentrations were tested in the present studies (data not shown). Differentiated PC12 cells were treated for 24 h with 0, 100, 200, or 500 *μ*M H_2_O_2_ with or without ERW. ERW significantly suppressed cell death when treated with 100 or 200 *μ*M H_2_O_2_ (Figure 1(a), ^*^ *P* < 0.05) but not with 500 *μ*M H_2_O_2_. SFME cells were treated for 24 h with 0, 50, 100, or 200 *μ*M H_2_O_2_ with or without ERW. ERW significantly suppressed cell death when treated with 50 or 100 *μ*M H_2_O_2_ (Figure 1(b), ^*^ *P* < 0.05) but not with 200 *μ*M H_2_O_2_. When these cell lines were treated with ERW alone (0 *μ*M H_2_O_2_) the number of viable cells did not increase compared to that of the controls (Figures 1(a) and 1(b)). Therefore, the results indicate that ERW suppresses neuronal cell death caused by H_2_O_2_-induced oxidative damage.

### 3.2. ERW and Antioxidants Protect Neuronal Cells from H_2_O_2_-Induced Cell Death

**Figure 2:**
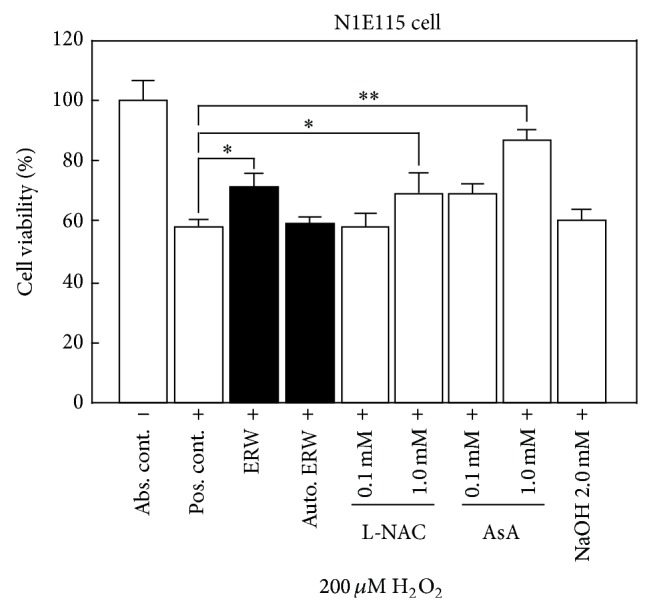
Protective effect of ERW against H_2_O_2_-induced cytotoxicity on N1E115 cells. N1E115 cells were preincubated with FBS/DMEM medium and were treated with serum-free medium made of MQ-water, ERW, L-NAC (0.1, 1 mM), AsA (0.1, 1 mM), or 2 mM NaOH supplemented with or without 200 *μ*M H_2_O_2_ for 24 h. A WST-8 kit was used to measure viable cell numbers at 450 nm using a microtiter plate reader. Data are expressed as mean ± standard deviation (SD) for three independent experiments. ^*^ *P* < 0.05, ^**^ *P* < 0.01. Abs. cont.: absolute control, Pos. cont.: positive control, Auto. ERW: autoclaved ERW.

We decided to further confirm ERW efficacy by using the known antioxidants, L-NAC and AsA as system controls. The cell viability for the absolute control was set as 100% (Figure 2). The cell viability for the positive control was reduced to 60% compared to the absolute control (Figure 2). When the N1E-115 cells were cultured with the medium made of ERW containing 200 *μ*M H_2_O_2_, the cell viability was significantly increased compared to the positive control (Figure 2, ^*^ *P* < 0.05) in agreement with the previous result [26]. We also found that autoclaved ERW was not protective against 200 *μ*M H_2_O_2_ induced cell death (Figure 2). When N1E-115 cells were cultured with the positive control medium supplemented with the known antioxidants, 1 mM L-NAC or 1 mM AsA, the viability increased significantly, compared with the positive control, respectively (Figure 2, ^*^ *P* < 0.05, ^**^ *P* < 0.01), and indicated the assay system was working properly. Cell viability was not affected when the cells were cultured with the positive control medium supplemented with 0.1 mM L-NAC or AsA or 2 mM NaOH (Figure 2). From the results, ERW was confirmed to exert protective effects against H_2_O_2_ induced cell death.

### 3.3. ERW Maintains Differentiated Morphology against H_2_O_2_-Induced Oxidative Stress

**Figure 3:**
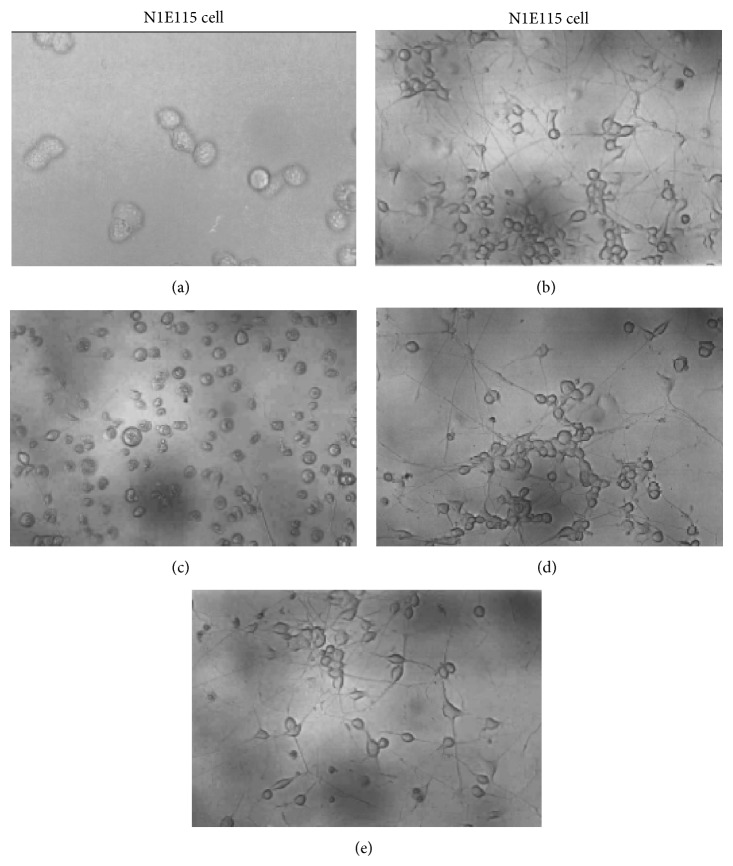
Protective effect of ERW on morphological changes against H_2_O_2_-induced oxidative stress on N1E115 cells. N1E115 cells were preincubated with FBS/DMEM medium and treated with serum-free medium made of MQ-water as the control (a), differentiated cells with DMSO (b), differentiated cells treated with 200 *μ*M H_2_O_2_ (c), differentiated cells treated with ERW alone (d), and differentiated cells treated with ERW and 200 *μ*M H_2_O_2_ (e) for 24 h. Morphological changes were observed microscopically and photographed.

We next tested whether ERW affects N1E-115 cell morphology. Undifferentiated cells exhibited a typical round shaped morphology (Figure 3(a)) while differentiated cells showed a neuronal network representing neurite outgrowth (Figures 3(b) and 3(d)). These cellular morphologies are quite similar to those of published data [33]. When the differentiated cells were cultured in the medium made of MQ-water containing 100 *μ*M H_2_O_2_, the neuronal network disappeared (Figure 3(c)) while the cells cultured with the medium made of ERW containing 100 *μ*M H_2_O_2_ maintained the original state of differentiated morphology displaying a neuronal network and neurite outgrowth (Figure 3(e)). These results demonstrated that ERW is capable of maintaining the morphological integrity of differentiated neuronal cells against H_2_O_2_-induced oxidative stress.

### 3.4. ERW Scavenges Intracellular ROS

**Figure 4:**
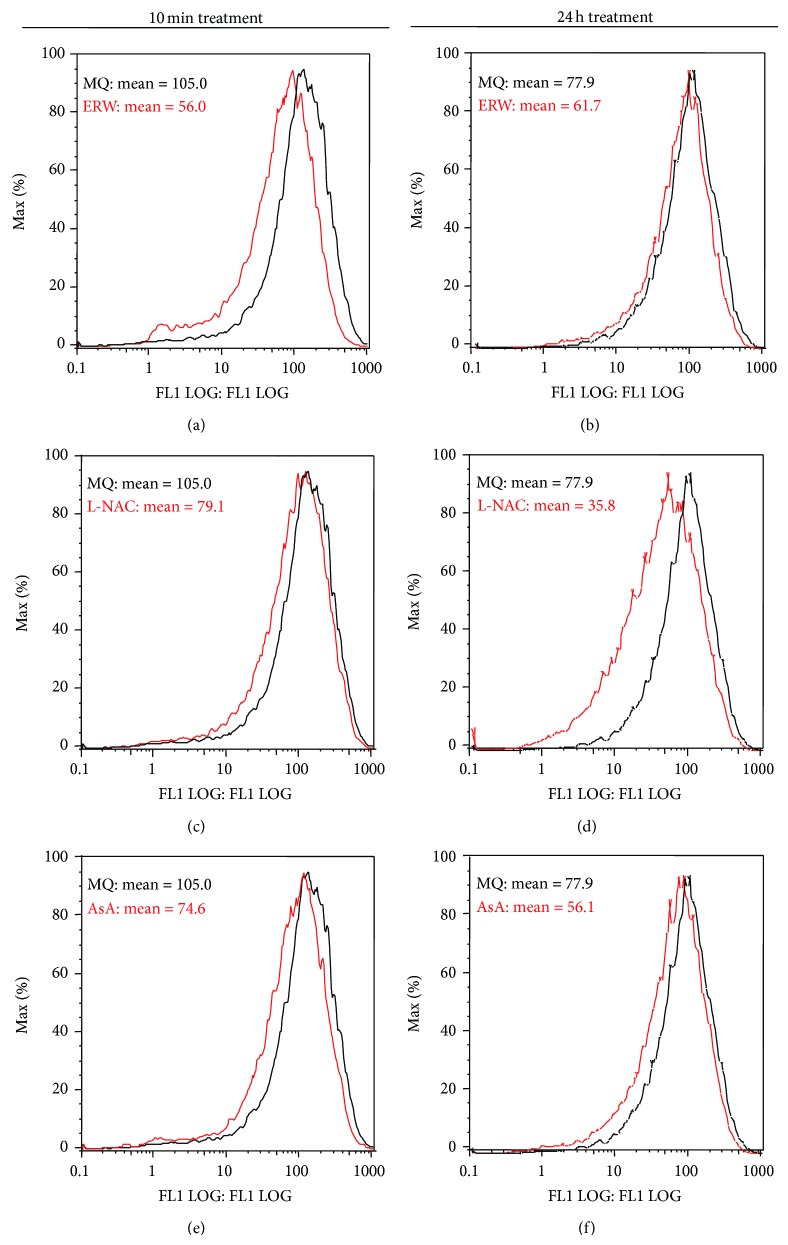
Flow cytometric analysis of the intracellular H_2_O_2_ scavenging ability of ERW on N1E115 cells. N1E-115 cells (7.5 × 10^4^ cells) were treated with ERW, L-NAC (1.0 mM), AsA (1.0 mM), or control MQ-water, and incubated for 10 min ((a), (c), and (e)) or 24 h ((b), (d), and (f)). After each treatment, cells were harvested and resuspended in 1 ml PBS. Fluorescence intensities were measured immediately using an EPICS XL System II-JK flow cytometer with excitation and emission wavelengths of 495 and 525 nm, respectively. Histograms were analyzed by using FlowJo software provided with the cytometer.

The results obtained thus far demonstrated the substantial protective effects of ERW on N1E-115 cells against H_2_O_2_-induced cell death. We examined whether ERW is protecting cell death by scavenging intrinsic intracellular ROS. The intracellular H_2_O_2_ level of the N1E-115 cells treated for 10 min with ERW (mean value = 56.0) was substantially decreased compared with the MQ-water control (mean value = 105.0) (Figure 4(a)). The intracellular H_2_O_2_ level of the cells treated for 24 h with the ERW (mean value = 61.7) decreased to a lesser degree from the MQ-water control (mean value = 77.9) (Figure 4(b)). When the N1E-115 cells were treated for 10 min with the medium containing 1 mM L-NAC or AsA, the leftward shift indicative of the intracellular H_2_O_2_ scavenging was small but clearly recognizable (Figures 4(c) and 4(e)). While the intracellular H_2_O_2_ level of 1 mM L-NAC treated cells for 24 h was substantially reduced compared to MQ-water control (Figure 4(d)), the scavenging effect of 1 mM AsA was evident but to a lesser extent (Figure 4(f)). Although the same experiment was performed with 0.1 mM of L-NAC and AsA, this concentration did not show an appreciable scavenging effect after either 10 min or 24 h treatment (data not shown). From these results, ERW was shown to scavenge intracellular H_2_O_2_ within a short time, while the scavenging capabilities of AsA, in particular L-NAC, are exerted for at least 24 hours.

**Figure 5:**
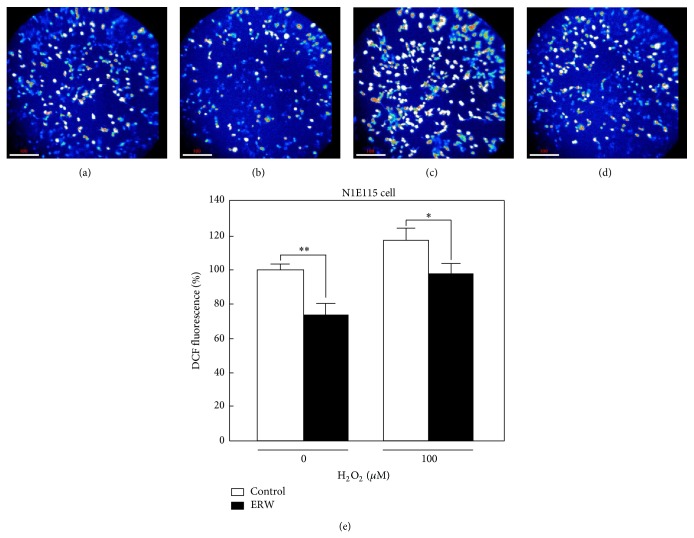
H_2_O_2_-induced ROS scavenging ability of ERW on N1E115 cells. Differentiated N1E115 cells were treated for 10 min with ERW with or without 200 *μ*M H_2_O_2_ and intracellular H_2_O_2_ levels were detected with a DCFH-DA probe and measured using a confocal laser microscope. Representative photographs of three independent experiments are shown: (a) MQ control, (b) ERW, (c) MQ + 100 *μ*M H_2_O_2_, and (d) ERW + 100 *μ*M H_2_O_2_. (e) Photographs were used to calculate intracellular fluorescence intensities reflecting H_2_O_2_ levels in the cells for each treatment. Data are expressed as mean ± standard deviation (SD) for three independent experiments. ^*^ *P* < 0.05 and ^**^ *P* < 0.01.

To confirm the flow cytometric data, we further tested the scavenging ability of ERW against induced intracellular H_2_O_2_ by using a combination of a DCFH-DA probe and a confocal microscope. Fluorescence intensities reflecting intracellular H_2_O_2_ levels obtained from the cells treated for 10 min with MQ-water alone (control) were set to 100% (Figures 5(e) and 5(a)). When cells were treated for 10 min with ERW alone, the fluorescence intensity was decreased approximately 30% compared with that of the MQ-water control (Figures 5(e) and 5(b), ^*^ *P* < 0.05). When the cells were cultured with the medium made of MQ-water containing 200 *μ*M H_2_O_2_, the intracellular H_2_O_2_ level was increased by approximately 20% compared with that of the MQ-water control (Figures 5(e) and 5(c)). When the cells were cultured with the medium made of ERW containing 200 *μ*M H_2_O_2_, the intracellular H_2_O_2_ level was suppressed approximately 20% compared with that of the MQ-water containing 100 *μ*M H_2_O_2_ (Figures 5(e) and 5(d), ^*^ *P* < 0.05). Representative images of 3 independent experiments are shown in Figures 5(a)–5(d). These images were used to generate Figure 5(e) by measuring the fluorescence intensities of each treatment. The same conclusion, ERW scavenges physiologically intrinsic H_2_O_2_ and exogenously induced H_2_O_2_, was reached from two independent detection methods.

### 3.5. Suppressive Effect of ERW on Nitric Oxide (NO) Toxicity in PC12 Cells

**Figure 6:**
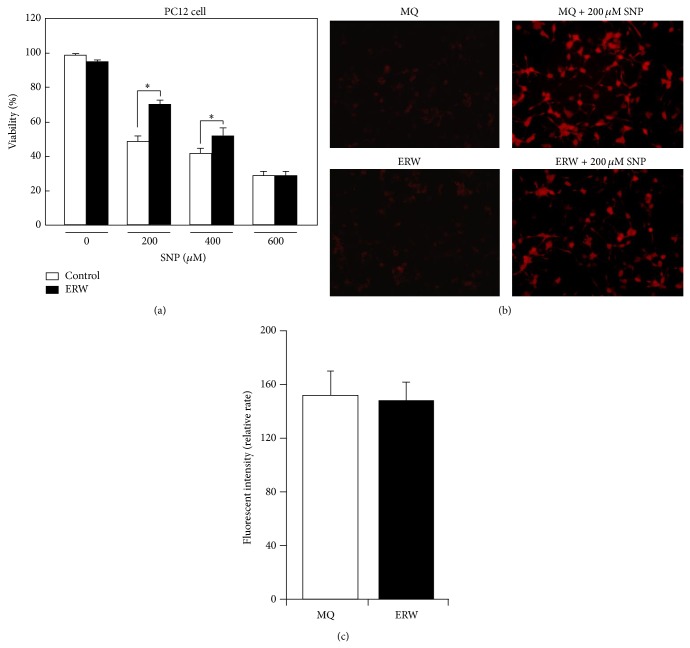
Suppressive effect of ERW on SNP-induced NO toxicity in PC12 cells. Cells were cultured for 24 h with the medium containing ERW or MQ-water each supplemented with 0, 200, 400, and 600 *μ*M SNP. (a) Cell viability was measured by a WST-8 kit. Data are expressed as mean ± standard deviation (SD) for three independent experiments. ^*^ *P* < 0.05. (b) ERW was assessed for its intracellular NO scavenging ability using the DAR-4M AM bioimaging probe. Representative photographs of NO dependent fluorescence levels incubated with MQ, ERW, and 200 *μ*M SNP added-MQ and ERW are shown. (c) Fluorescence intensities of 50 cells treated with MQ or ERW containing 200 *μ*M SNP were measured and Tukey's multiple comparison method was used to compare the effect of MQ and ERW.

In the MQ-water control medium, SNP reduced PC12 cell viability in a manner approximating dose-dependency (Figure 6(a)). However, when cultured in the medium made of ERW, the cell viability was restored significantly, compared with the respective controls at 200 and 400 *μ*M SNP (Figure 6(a), ^*^ *P* < 0.05). Thus, ERW could restore cell viability against NO generated by up to 400 *μ*M SNP. However, this finding does not prove that ERW is directly scavenging NO. To clarify this point, we assessed the intracellular NO level directly using the NO-specific DAR-4M AM probe. Differentiated PC12 cells were treated with the medium made of ERW or MQ-water with or without 200 *μ*M SNP. After the SNP treatments, NO-induced DAR-4M intracellular fluorescence was observed by a fluorescence microscope and photographed (Figure 6(b)). Intracellular fluorescence was barely visible in the cells cultured with the medium made of either MQ-water or ERW (Figure 6(b), left side panels designated as MQ and ERW). Intracellular red fluorescence indicative of SNP induced NO was evident in the cells cultured with the medium made of MQ-water and ERW (Figure 6(b), right side panels designated as MQ + 200 *μ*M SNP and ERW + 200 *μ*M SNP). Using two of the right side panels, the fluorescence intensities were scored for 50 cells randomly picked in each panel. Analysis revealed no statistically significant differences between MQ- and ERW-treated cells (Figure 6(c)). Thus, ERW was found to lack direct NO scavenging activity in PC12 cells.

### 3.6. Suppressive Effect of ERW on Glutamate-Induced Ca^2+^ Influx and Cell Death

**Figure 7:**
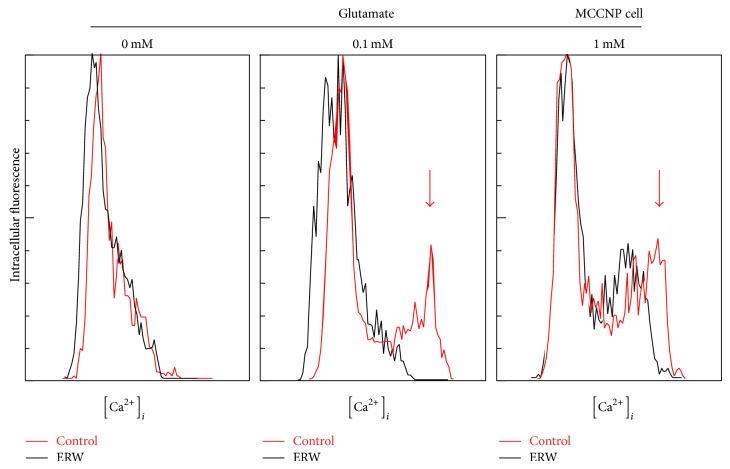
Suppressive effect of ERW on glutamate-induced Ca^2+^ influx in MCCNP cells. Intracellular Ca^2+^ was measured with Fluo-3 AM, a membrane-permeable calcium sensitive dye. Internalized Fluo-3 AM is hydrolyzed by intracellular esterases liberating Flou-3 which can react with intracellular free Ca^2+^ ions forming a fluorescent complex. After pretreatment with glutamate (0, 0.1 or 1.0 mM) in the medium made with ERW or MQ-water for 15 min, MCCNP cells were stained with Fluo-3 AM for 20 min and post-incubated with FBS/HBSS medium. A fluorescence microscope was used to detect intracellular fluorescence and the images were recorded by a digital camera. Digital images were converted to numerical data with the aid of the NIH image analysis program and then analyzed by Excel software. Representative histograms of triplicate independent experiments are shown.

**Figure 8:**
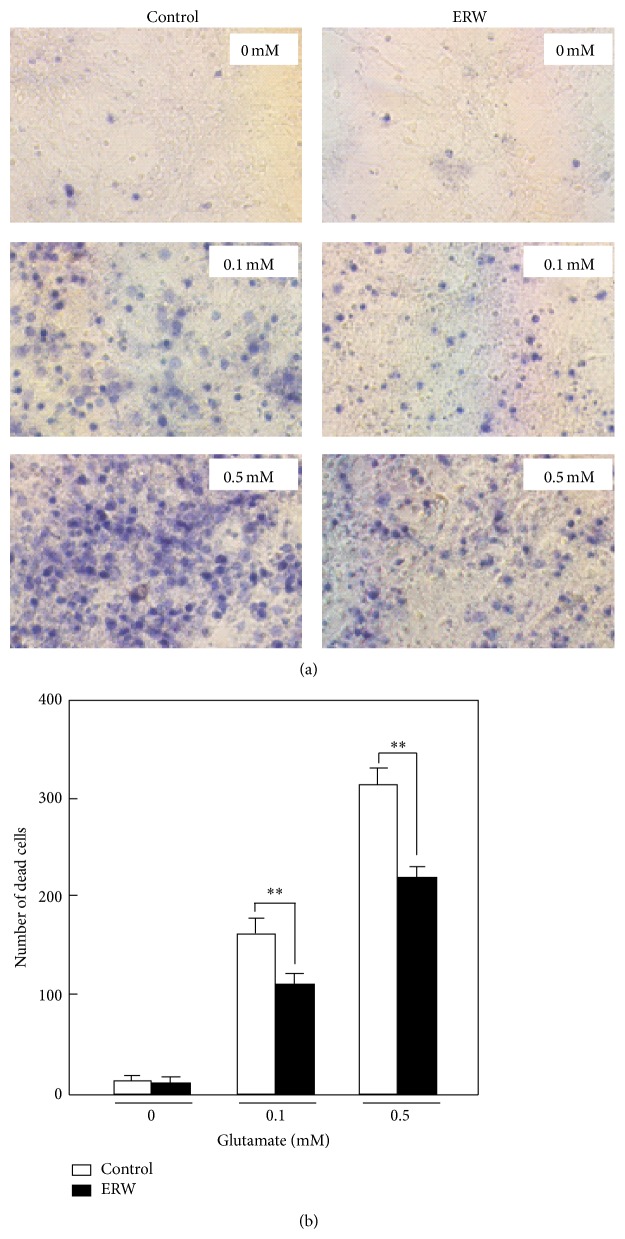
Protective effect of ERW against glutamate toxicity on MCCNP cells. After MCCNP cells were treated with 0, 0.1, and 0.5 mM glutamate in the medium made of ERW or MQ-water for 24 h, nonviable cells were stained with trypan blue dye. Nonviable cells stained blue were counted for 8 randomly selected fields as one set and 4 sets for each treatment were counted under the microscope. Representative photographs are presented in (a) from which stained cell counts were statistically analyzed as shown in (b). Data are expressed as mean ± standard deviation (SD) for four independent experiments. ^**^ *P* < 0.01.

We used MCCNP cells to examine whether ERW can affect Ca^2+^ influx. As shown in Figure 7, the MQ-water control (red line) treated cells showed a new Ca^2+^ peak as indicated by a downward arrow in the 0.1 and 1 mM glutamate treated cells compared with the 0 mM glutamate treated cells. ERW (black line) in the presence of 0.1 mM glutamate treated cells did not show such a peak indicative of Ca^2+^ influx. Even though the 1 mM glutamate treated cells cultured in the presence of ERW showed a peak, the calcium concentration [Ca^2+^]_i_ is less than that of the control. The results indicate that ERW suppresses glutamate induced Ca^2+^ influx in the MCCNPcells which suggests a reduction of cell death. This possibility was further tested using the trypan blue exclusion assay. As shown in Figure 8(b), dead cells treated with the medium made of the MQ-water control with 0.1 and 0.5 mM glutamate increased in a dose-dependent manner compared to those grown without glutamate. Similarly, a dose-dependent increase in dead cells was observed when the cells were treated with the medium made of ERW containing 0, 0.1 or 0.5 mM glutamate. However, the number of dead cells grown in the medium with ERW containing 0.1 or 0.5 mM glutamate was significantly reduced compared with those grown in the presence of the respective MQ-water controls (Figure 8(b), ^**^ *P* < 0.01). The representative photographs are shown in Figure 8(a). The results indicate that ERW suppresses glutamate-induced cell death which correlated well with the suppression of Ca^2+^ influx.

## 4. Discussion

In the present study, ERW was confirmed to suppress H_2_O_2_-induced cell death in N1E-115, PC12 and SFME cell lines. Further studies with N1E-115 cells demonstrated that the suppressive effect of ERW stemmed from its ROS scavenging ability. This finding is in accordance with the previous reports obtained from pancreatic *β*-cell HIT-T15 cells [15, 19], human lung carcinoma A549 cells [41], HT1080 tumor cells [24], and diabetic* db/db* mice [48].

**Figure 9:**
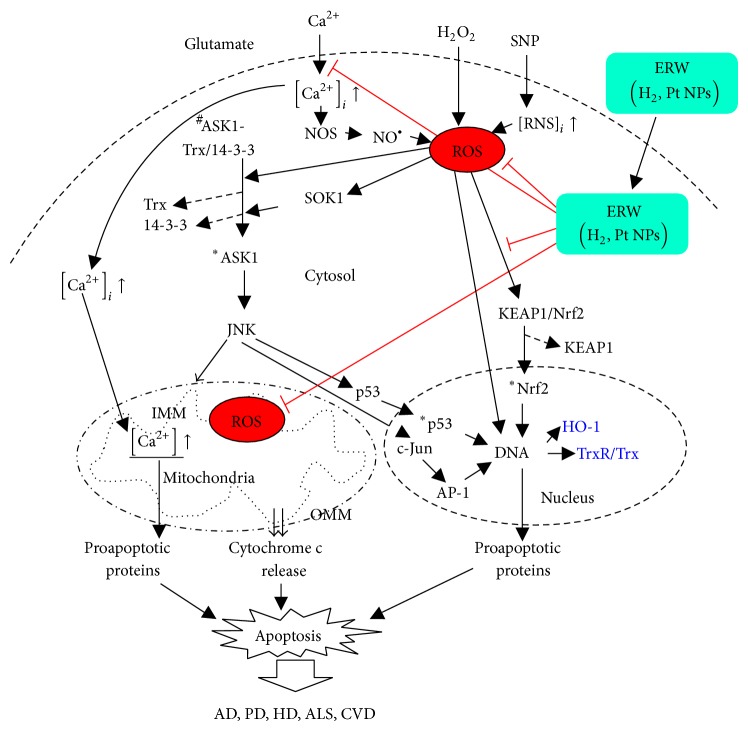
Schematic representation of possible points of action for the suppressive effects of ERW against stressors. Glutamate induces Ca^2+^ influx leading to increased neuronal cell death. SNP increases neuronal cell death by inducing NO. A reduction of neuronal cells eventually leads to NDs. ERW suppresses Ca^2+^ influx and scavenges ROS, thereby protecting neuronal cell death. Arrows indicate a single step or multiple intermediary steps responsible for upregulating the proapoptotic genes triggered by ROS, RNS, and Ca^2+^. HO-1 and TrxR/Trx, which are controlled by vitagenes, act as homeostasis keepers within a tolerable stress zone and are included in blue font. ^#^ indicates inactive state. ^*^ indicates activated state. (↑) is increased intracellular Ca^2+^ and RNS levels. Symbol (⊢) with red color represents prospective points of action of ERW.

The current study further revealed that ERW significantly suppresses cell death caused by glutamate- or SNP-induced stresses although it does not have direct NO scavenging activity. We pursued further experiments to unravel the mechanism underlying this phenomenon. Glutamate toxicity is mainly mediated by the glutamate receptor, NMDA, involving Ca^2+^ influx into cerebral cortex cells in the CNS [49]. Internalized Ca^2+^ and endogenous calmodulin together activate neuronal NO synthase for the production of NO. Importantly, neuroprotective NO is converted to neurodestructive NO radical (NO^•^) depending on the redox condition in the neuronal cells [11]. Following NO^•^ production, it reacts with O_2_^•−^ to form ONOO^−^ which decomposes to nitrite and highly reactive HO^•^, and the reactions proceed to generate stable nitrite/nitrate species. Such radicals liberated during the NO-cascade contribute to neurocytotoxicity [2, 10–12, 50]. Based on the information for Ca^2+^ influx induced by glutamate in cerebral cortex cells [49] and the NO-cascade as discussed above, we used MCCNP cells to assess whether ERW scavenges NO. Firstly, we found that ERW clearly suppressed Ca^2+^ influx in MCCNP cells and ERW suppressed extracellular H_2_O_2_ induced Ca^2+^ influx in PC12 cells (data not shown). Also, a previous study demonstrated that ERW prevents alloxan-induced elevation of intracellular Ca^2+^ levels in HIT-T15 *β*-cells [19]. Moreover, exposure of rat mixed cortical neuron primary cells to H_2_O_2_ resulted in increases in intracellular Ca^2+^ levels which are suggested to be involved in H_2_O_2_-induced cell death [51]. Subsequently, H_2_O_2_-induced neuronal cell death was shown to be closely associated with NMDA mediated Ca^2+^ influx [52]. The present finding of the suppressive effect of ERW is intriguing because increased Ca^2+^ influx triggers calpain (a protease) activation leading to the cleavage of a neuron specific p35 activator protein to p25 which binds to cyclin dependent kinase 5 (Cdk5) forming a hyperactive Cdk5/p25 complex. This deregulated Cdk5/p25 activity has been linked to be a causative factor of various neurodegenerative diseases [6]. Secondly, we used the trypan blue exclusion assay to evaluate the protective function of ERW on the cytotoxic effect induced by glutamate in MCCNP cells. ERW significantly suppressed glutamate induced cell death which is closely correlated with reduced Ca^2+^ influx. Finally, we performed experiments using SNP and PC12 cells in combination with the NO-specific DAR-4M AM probe to determine whether ERW scavenges NO directly. We utilized PC12 cells because this cell line has been described to be sensitive to NO [38]. To our surprise, ERW was not found to scavenge NO; nevertheless, the cell viability was significantly increased. In addition, in the presence of ERW, we did not observe the reduction of intracellular fluorescence intensities at SNP concentrations ranging from 50 to 500 *μ*M (data not shown). SNP has been used as a NO-donor [30, 49, 50] and its metabolites have been suggested to be involved in redox cycling with oxygen to form O_2_^•−^, which then produces H_2_O_2_, which in turn produces HO^•^ via the Fenton reaction [30]. Therefore, the presently observed suppressive effect of ERW on SNP induced PC12 cell death is mostly caused by its ROS scavenging ability and it is less likely that it can scavenge NO directly. The proposed points of action of ERW are shown in Figure 9.

The question remains as to what factors are responsible for the presently observed scavenging activity of ERW against ROS produced by glutamate, SNP, and H_2_O_2_. ERW has been shown to contain dissolved hydrogen (0.4–0.9 ppm) and Pt nanoparticles (nps) (0.1–2.5 ppb) as potential ROS scavenging substances [15–17]. Recently, we estimated the levels of dissolved hydrogen to be 0.76 ppm in freshly prepared ERW with a half-life of approximately 100 min [24]. Direct involvement of hydrogen molecules as a ROS scavenger was reported using rat myotube L6 cells [53]. Also, hydrogen molecules were shown to enhance the expression of genes for antioxidant enzymes such as catalase, glutathione peroxidase, and heme oxygenase [53]. In another report, ERW was shown to restore the activities of superoxide dismutase, catalase, and glutathione peroxidase in mice, supporting the idea that ERW acts as an antioxidant and an ROS scavenger [25]. Similarly, an antioxidative role of ERW was reported* in vitro* and* in vivo* [54–56]. ERW was also reported to possess reducing activities because of the presence of dissolved molecular hydrogen [57, 58].* In vivo*, 0.08 ppm of hydrogen molecules were reported to be sufficient to exert antineurodegenerative effects in PD model mice [59]. Also, hydrogen molecules were shown to reduce ^•^OH, ONOO^−^ and oxidative stress in the rat brain induced by focal ischemia and injury [60]. Recently, molecular hydrogen dissolved in ERW derived from tap water was reported to reduce LPS-induced neuroinflammation in mice [61]. Based on these studies, it is highly probable that hydrogen molecules in ERW can reach and deliver a neuroprotective effect in the brain.

Another potential factor which plays a role in the ROS scavenging activity of ERW is the Pt nps derived from the Pt-coated Ti electrodes during electrolysis. Comparative measurements using an inductively coupled plasma mass spectrometer (ICP-Ms) have shown that Pt nps and related compounds are detected only in the post-electrolyzed 2 mM NaOH solution, that is, ERW, and not in the preelectrolyzed NaOH solution [15, 17]. Furthermore, Pt nps in several ERW preparations were measured to range from 0.1 to 2.5 ppb using ICP-MS [15–17, 21]. The variable amount of Pt nps in different ERW preparations was thought to result from the gradual erosion of Pt coated Ti-electrodes from repeated electrolysis [24]. It was also necessary to confirm if Pt nps can scavenge ROS. In this respect, it has been shown that synthetic Pt nps can directly scavenge O_2_^•−^, HO^•^, and H_2_O_2_ and are considered to be a multifaceted ROS scavenger [17, 62]. In agreement with our results, synthetic Pt nps have been reported to show ROS scavenging activity [63–65]. Moreover, it is noteworthy to mention that the liberated Pt nps generates active hydrogen from the hydrogen molecules simultaneously produced during electrolysis. In turn, the active hydrogen is adsorbed/absorbed to nearby Pt nps which may synergistically contribute to the scavenging effect of Pt nps [62, 66]. We tested whether autoclaving will affect the antioxidative ability of ERW which resulted in the loss of the protective effect against H_2_O_2_-induced N1E-115 cell death. Similar results were reported in other cell lines and protecting factors have been suggested to be heat unstable or volatile substances contained in the ERW [19]. It has been reported that synthetic Pt nps will agglomerate after autoclaving leading to inefficient cellular uptake and dissolved hydrogen molecules and active hydrogen adsorbed/absorbed in the Pt nps are thought to be forced out [24, 62]. Therefore, autoclaving of ERW provides further support that it contains hydrogen molecules and Pt nps as active substances. For ERW to be useful for* in vivo* therapeutic applications, Pt nps must be able to reach brain cells. Following ingestion, Pt nps are required to survive several obstacles including intestinal cell wall penetration, blood flow, and the BBB before reaching the brain cells. Pt nps were shown to be taken up by cells via mainly pinocytosis [62, 67, 68]. Another critical obstacle which will limit the usefulness of Pt nps is their ability to cross the BBB as has been suggested for other antioxidants [2, 6]. Several diseases including AD leads to reduced BBB integrity [69] suggesting that Pt nps has the opportunity to pass through the diseased BBB more easily than the healthy BBB to gain access to the brain cells. A recent article identifies the hurdles associated with synthetic metal nps delivery to the brain [69]. Therefore, further* in vivo* studies are required to investigate Pt nps containing ERW as suggested for other metal nps [62, 69–71].

ROS have been regarded as toxic by-products of physiological metabolism and increased ROS generation and reduced mitochondrial membrane potential (ΔΨm) are closely associated with the oxidative stress in PC12 cells [72]. In the present study, ERW was found to protect 3 neuronal cell lines from direct oxidative damage induced by H_2_O_2_ implying that ERW protects mitochondria in neuronal cells. However, further investigation using N1E-115 cells treated with ERW/H_2_O_2_ (200 *μ*M) medium did not show significant restoration of ΔΨm compared with the MQ/H_2_O_2_ (200 *μ*M) medium treated control (data not shown).

There are multiple pathways that induce cell apoptosis. Among these, ROS mediated pathways involving mitochondria are relevant to the results obtained in this study. ROS induced oxidative stress causes oxidative damage to vital cellular constituents, which eventually affect cell viability. ROS accumulation in cells mediates apoptosis by way of mitochondria-dependent and mitochondria-independent pathways. Elevated ROS levels in the cell will disturb the physiological balance maintained by the proapoptotic (for example Bax, Bak, Bok, Bid, and Bim) and antiapoptotic (for example Bcl-2, Bcl-X_L_, and Bcl-w) proteins, all of which belong to the Bcl-2 family of proteins and generally act to induce apoptosis or survival of the cell. Apoptosis is induced when proapoptotic Bcl-2 family proteins (Bax, Bak, Bok, Bid, and Bim) are activated by death signals via death receptors (tissue necrosis factor receptor-1 (TNFR1), Fas, and TRAIL-R1). Tissue necrosis factor-*α* (TNF*α*) increases the ROS level, which in turn activates the apoptosis signal regulating the kinase 1-c-Jun NH_2_ terminal kinase (ASK1-JNK) apoptotic pathway. Exogenously and endogenously derived ROS are considered the most effective activators of ASK1, which acts as a redox sensor because it contains the redox-sensitive regulatory protein thioredoxin (Trx) binding domain. ROS inactivates Trx by oxidizing two cysteine residues in the redox center of Trx causing it to dissociate from ASK1. This allows ASK1 to be bound by the tumor necrosis factor receptor-associated factor 2 (TRAF2) and TRAF6 to form an active ASK1 signalosome that induces the autophosphorylation of ASK1. During the ASK1 activation process under oxidative stress, the 14-3-3 proteins binding site at Ser-966 of ASK1 is dephosphorylated resulting in the dissociation of 14-3-3 proteins which are facilitated by the ROS activated mammalian sterile 20 (Mst) family member SOK-1. The ROS activated ASK1 eventually activates the JNK cascade and sustained JNK activity leads to apoptosis. Thus, oxidative stress activates ASK1 which phosphorylates the MAPKK-JNK and -p38 pathways. Activated JNK directly phosphorylates p53 rendering it to translocate to the nucleus, allowing up-regulation of proapoptotic genes such as the p53 upregulated modulator of apoptosis (PUMA) and BCL2-associated X protein (Bax). Alternatively, the phosphorylated JNK relocates to the nucleus and phosphorylates c-jun to form activator protein 1 (AP-1) resulting in the up-regulation of proapoptotic genes such as TNF*α*, Fas-L, and Bak. Moreover, activated JNK moves to the nearby mitochondria and binds to the complexes of proapoptotic Bax and tBid produced from proapoptotic Bid by active caspase-8 and then tBid with Bax/Bax and Bak/Bak oligomers initiate pore formation in the outer mitochondrial membrane (OMM) causing the release of cytochrome c. Additionally, other factors including AIF, EndoG, and CAD, which relocate to the nucleus for DNA fragmentation, and the proapoptotic protein second mitochondrial activator of the caspase/direct inhibitor of the apoptosis protein binding protein with low pI (Smac/DIABLO) and mammalian serine protease (HtrA2/Omi) induce apoptosis via indirect activation of caspase-8 apoptotic pathways. Released cytochrome c forms an apoptosome with Apaf-1 and procaspase-9 resulting in the formation of active caspase-9, which activates the executioner caspase-3. Separately, caspase-3 gets activated by the active caspase-8. Activated caspase-3 executes cell destruction by cleaving various vital constituents in the cells. Caspases are constitutive residents of the cytosol as inactive zymogen monomers and are activated for example by endogenously and exogenously generated H_2_O_2_ through oxidation of the cysteine catalytic sites. Exposure to H_2_O_2_ decreases the mitochondrial transmembrane potential (ΔΨm) and oxidizes the mitochondrial permeability proteins comprising voltage dependent anion channel (VDAC), adenine nucleotide translocase (ANT), and cyclophilin D (CyD) leading to mitochondrial Ca^2+^ influx. Thus, reduced ΔΨm, elevated Ca^2+^ levels, and oxidative stress induce enlargement of the mitochondrial permeability transition pore (MPTP) located at the inner-outer membrane contact site to release cytochrome c. Elevated ROS will damage nuclear DNA directly and, in particular, H_2_O_2_ stimulates the nuclear accumulation of Forkhead box O3 (FOXO3) transcription factor, which transactivates the Bcl-2 interacting mediator of cell death (Bim) and other genes. Activated Bim is then translocated to the mitochondria causing a ROS burst via respiratory chain uncoupling. At the intermitochondrial membrane (IMM), this and other events of proapoptotic protein activation induce OMM perforation in a Bax/Bak dependent-manner[73–77]. Therefore, elevated ROS affects multiple sites that drive the cells toward apoptosis. The present results can be used to elucidate the possible ERW action sites contributing to the increased cell viability. ERW contains hydrogen molecules and Pt Nps as potential functional components as described above. Dissolved hydrogen molecules diffused in cells have been shown to reduce intracellular hydroxyl radicals [78, 79]. Pt Nps have been shown to be taken up by pinocytosis, diffusion, and other processes and act as novel ROS scavengers* in vitro* and* in vivo* [80, 81]. Internalized functional components are capable of scavenging ROS produced by cytotoxic stimuli such as H_2_O_2_, SNP, and glutamate used in this study. Based on the pathways mentioned above, ERW could scavenge ROS to prevent thioredoxin (Trx) inactivation and inhibit SOK1 activation, thereby preventing active ASK1 signalosome formation. An inactive ASK1 signalosome becomes incapable of forwarding the ASK1-JNK pathway and prevents subsequent apoptosis prone signals including cytochrome c rerelease, p53 activation, and AP-1 formation. H_2_O_2_ scavenging prevents intracellular Ca^2+^ accumulation. Moreover, scavenging H_2_O_2_ is likely to prevent the nuclear accumulation of FOXO3 that subsequently induces mitochondrial ROS burst. The present results of reduced ROS and [Ca^2+^]_*i*_ levels cooperatively contribute to improve the mitochondrial integrity leading to the increased cell viability. At present, the mechanism by which ERW functioned to reduce the [Ca^2+^]_*i*_ level is not clear and further studies are required. The proposed points of action of ERW on cellular stressors are shown in Figure 9.

In the present study, the cell viability was found to be significantly improved because of the ROS scavenging ability of ERW. It is known that excessively produced ROS lead to oxidative stress and neurodegenerative diseases. The present study simulated excess ROS/RNS levels by applying H_2_O_2_, glutamate, and SNP to the cells. Recent reports emphasize that cells that possess a group of genes termed vitagenes have a better ability to maintain cellular homeostasis under various stress conditions. Based on the hormetic concept, low doses of oxidative stresses may induce cellular defense pathways to mediate a hormetic adaptive response conferred by the expression of vitagenes coding for stress resistance proteins that maintain cellular homeostasis. Vitagenes include the genes coding for the heat shock proteins (HSP) such as Hsp32 (also called Heme oxygenase 1, HO-1) and Hsp70, sirtuins, and the thioredoxin/thioredoxin reductase systems. Under oxidative conditions, the Hsps protects brain cells by degrading the prooxidant heme to free iron, carbon monoxide, and biliverdin, which in turn is reduced by biliverdin reductase to bilirubin, which possess endogenous NO and RNS scavenging abilities. Conversely, excess bilirubin production becomes neurotoxic by way of cell membrane disruption, mitochondrial transmembrane potential (ΔΨm) reduction, and apoptotic pathway activation. Thus, HO-1 repression is important for cell survival as excess bilirubin is cytotoxic. The heme oxygenases function as oxidative stress sensors and modulators of redox homeostasis. SIRT1 (silent information regulator 2 (Sir2) protein 1) is a sirtuin possessing histone deacylase activity and it plays a role in the hormetic response of cells to oxidative stressors. Sirtuins in the presence of NAD^+^ as a cofactor deacylate transcription factors such as Nrf2, p53, FOXO (Forkhead box O), and nuclear factor-*κ*B (NF-*κ*B). H_2_O_2_ is known to upregulate SIRT2 expression and it binds to FOXO3a enhancing its binding to target genes such as MnSOD. Thus, increased SIRT1 and SIRT2 expression contributes to ROS reduction. Also, the FOXO subfamily of transcription factors transactivates the Bcl-2 interacting mediator of cell death (Bim) and other genes. Another vitagene system is the thioredoxin/thioredoxin reductase (Trx and Trx reductase) system. Trx exists as a cytoplasmic protein (Trx-1) and a mitochondrial (Trx-2) protein essential for cell survival. It regulates cellular redox balance by reversible oxidation of the two cysteine residues in the redox center of Trx. Trx acts as an antioxidant or ROS scavenger as it eliminates singlet oxygen (^1^O_2_), hydroxyl radical (OH^•^), and hydrogen peroxide (H_2_O_2_). H_2_O_2_ stimulates Trx-1 expression by the transcription factor Nrf2 and numerous stimuli including H_2_O_2_, hypoxia, and UV irradiation will direct translocation of Trx to the nucleus where it regulates the activities of critical transcription factors such as AP-1 members, NF-kB, p53, and Jun, which all are related to apoptosis pathways [82–87]. Vitagenes could function in preserving cellular homeostasis during stressful circumstances within a tolerable dose range, the hormetic zone. However, beyond this hormetic zone, higher degrees of oxidative stresses are thought to disrupt cellular redox homeostasis. In the present study, we monitored cell viabilities affected by various concentrations of H_2_O_2_, glutamate, and SNP. The results demonstrated that the cell viabilities were increased or recovered significantly except samples treated with the lowest and the highest concentrations of the stressors. From the results, it is possible that the reduced ROS induced by the presence of ERW in the cell could have modulated the oxidative states such that they fell again within the range of the hormetic zone, thereby allowing vitagenes to exert their activities.

## 5. Conclusion

ERW was found to protect N1E-115, PC12, SFME, and MCCNP cells from oxidative stresses caused by H_2_O_2_, glutamate, and SNP treatments. This neuronal cell protection stemmed from the ROS specific scavenging ability of dissolved hydrogen and Pt nps in the ERW. The present communication provides encouraging data for the therapeutic applicability of ERW against NDs.

## Abbreviations

AD: Alzheimer’s disease
ALS: Amyotrophic lateral sclerosis
Ara-C: Cytosine *β*-D-arabinofuranoside hydrochloride
AsA: Ascorbic acid
BBB: Blood brain barrier
CDL: Chemically defined lipid
CNS: Central nervous system
DAR-4M AM: Diaminorhodamine-4M acetoxymethyl ester
DCFH-DA: 2′, 7′-Dichlorofluorescin diacetate
DMSO: Dimethyl sulfoxide
ERW: Electrolyzed reduced water
HD: Huntington’s disease
OH•: Hydroxyl radical
HEPES: 4-[2-Hydroxyethyl]-1-piperazineethane-sulfonic acid
L-NAC: N-Acetyl-L-cysteine
NO: Nitric oxide
mEGF: Mouse epidermal growth factor
NOS: Nitric oxide synthase
${\text{NO}}^{•}$: Nitric oxide radical
NMDA: N-Methyl-D-aspartate
${\text{ONOO}}^{-}$: Peroxynitrite
PD: Parkinson’s disease
Pt: Platinum
RNS: Reactive nitrogen species
ROS: Reactive oxygen species
SNP: Sodium nitroprusside
${{\text{O}}_{2}}^{•-}$: Superoxide radical.
